# Impacts of Sea Level Rise on Danish Coastal Wetlands – a GIS-based Analysis

**DOI:** 10.1007/s00267-024-02096-9

**Published:** 2024-11-29

**Authors:** Paula Canal-Vergés, Lars Frederiksen, Sara Egemose, Torben Ebbensgaard, Kristian Laustsen, Mogens R. Flindt

**Affiliations:** 1 Department of Biology, University of Southern Denmark, Odense, Denmark; 2 COWI A/S, Parallelvej 2, 2800 Kongens Lyngby, Denmark; 3 Mariagerfjord Kommune, Adelgade 30B, 9500 Hobro, Denmark

**Keywords:** Climate change, Danish national Spatial plans, Coastal flooding, Coastal nature, Sea level rise, Coastal meadows, Salt marshes

## Abstract

Intergovernmental Panel on Climate Change (IPCC) scenarios run by an ensemble of models developed by the Coupled Model Intercomparison Project (CMIP) projects an average sea level rise (SLRs) of 0.6 to 1.2 m for the low and high emission scenarios (SSP1-1.9, SSP5-8.5), during the next century (IPCC 2021). The coastal zone will experience an increase in the flooding of terrestrial habitats and the depth of marine productive areas, with potential negative consequences for these ecosystems. The coast in Denmark is highly modified due to anthropogenic uses. Dikes, dams, and other coastal infrastructure are widespread, causing a coastal squeeze that prevents natural coastal development and inland migration of coastlines. We performed a national-scale analysis on the impacts of mean sea level rise (MSLR) in 2070 and 2120, and a 1 in 10-year storm surge water level (10SS) in 2120 MSLR for the Danish coast. Our study shows extensive permanent flooding of coastal habitats (~14%), whereas only 1.6% of urban areas will be flooded. Finally, very large agricultural areas (~191,000 ha) will be frequently flooded by 10SS if no extra protective measures are planned. With the present coastal protection structures, key habitats will be affected by permanent flooding or coastal squeeze while even larger extents will be subjected to intermittent marine flooding. About 45% (199 km^2^) of all Danish coastal wetlands will be permanently flooded by 2120, while areas occupied by forest, lakes and freshwater wetlands will be more frequently flooded by marine water. This study highlights the importance of including coastal habitats as dynamic elements in climate adaptation plans. Conservation and restoration of key habitats such as coastal wetlands should be prioritized in management plans. If Denmark does not change its current priorities, it may face the complete loss of coastal wetlands habitat in the 22nd century.

## Introduction

Climate change is projected to induce the rising of the mean sea levels, as well as increase the frequency of extreme sea levels due to storms (Storm surges, SS) (IPCC 2021). The natural habitats closest to the coast, among them coastal meadows, will be most affected. In a sea level rise due to global warming, depending on the local conditions, coastal wetland can either migrate or disappear. In Denmark, large budgets are spent on avoiding climate-related flooding as part of the climate adaptation plans of the municipalities.

Anthropogenic coastal protection (such as dikes and pumps) is widely prioritized in urban and rural areas, aiming to protect and conserve human infrastructure, industry, and agriculture areas. Coastal nature is widely neglected in these plans. Hence, Denmark’s coastlines are constantly changing, primarily because of human-made initiatives and secondarily due to natural forces. For instance, about 40,000 ha of the country, currently below sea-level, are kept dry through 930 embankments and dikes. This corresponds to ~20% of the Danish marine area with a depth of less than 2 m. Around 14% of the Danish coastline has disappeared as a result of these embankments, while 6% of the current coastline is estimated to be protected by dikes (Waagepetersen et al. 1986). In some places new land is reclaimed to the coast, while in other areas land is being eroded and lost to the sea. These changes have already created large local variations in water levels, storm impacts, currents, waves, and the overall distribution between land and water. Man-made measures such as dikes, locks, dams, and urban development in lowland areas induce changes in erosion and deposition patterns, ultimately altering the coastal landscape and affecting natural habitats. These changes affect important coastal habitats, such as coastal wetlands, which need certain hydrodynamic conditions, accretion processes and space to migrate when the sea level is rising.

In general, Denmark needs to reinforce and protect remaining key habitats in its landscape, which is largely dedicated to agriculture and urbanization, to maintain its biodiversity. To support this needed biodiversity in Danish Nature, several open nature types are protected through paragraph 3 of Denmark’s 1992 Nature Conservation Act (§3). The Act protects heaths, meadows, moors, bogs and marshes, coastal wetlands (coastal meadows and salt marshes) and beach swamps, grasslands, lakes, and streams as well as natural transitions between them when they individually or together are over 2500 m^2^.

In a parallel process, the European Union has sought to guide the environmental activities of member states. In response to these initiatives, Denmark has identified 261 Natura 2000 areas from which 257 are protected under the habitat directive and 124 under the bird directive, some of which coincide geographically with paragraph 3 (§3) protected areas. Natura 2000 areas cover 9% of the Danish land and 26% of the Danish Sea (Danish environmental protection agency, 2021). These areas are designated to protect endangered, vulnerable, or characteristic animals, birds, plants, and habitats, as stated in the individual ‘designation basis’ for each area. Natura 2000 habitats have a more restrictive definition, and regulation than §3 habitats. Paragraph 3 areas allow more flexibility in traditional land management without the formal requirements for environmental assessments seen in Natura 2000 areas, which prioritize biodiversity protection on a European scale.

The most recent national registration, in 2016, indicated that ~10.3% of the Danish territory is classified as §3-protected nature. However, a very small fraction of these protected area is classified as strictly protected, only 4% of the assigned marine areas and none of the terrestrial areas (Biodiversitetsrådet 2022). Denmark is thus, very far from the targets in the EU’s biodiversity strategy, where by 2030, 30% of the national territory should be protected nature, of which a third must be strictly protected (Biodiversitetsrådet 2022).

Coastal wetlands, in Denmark mostly coastal meadows and to a lesser coverage salt are important and highly biodiverse habitats. These wetlands are habitat for many birds, as breeding or migration areas for, e.g., waders, gulls, or terns and as foraging and resting areas for many waterfowl, waders and birds of prey. In addition, several internationally protected amphibians (EU habitat directive, Annex IV) are closely linked to (parts of) the meadows. Finally, coastal wetlands are important geophysical structures promoting coastal protection and sequestering of carbon.

Denmark hosts large areas of threatened European coastal meadows. The country contains 78.5% of the total European coastal meadow area in the continental zone and 14.5% in the Atlantic zone (EIONET 2022; data from 2013–2018). It has Europe’s second-largest extension of Atlantic coastal salt marsh (A2.5c, vulnerable) and fourth-largest extension of Baltic coastal meadow (A2.5b, endangered), respectively (European Commission, Directorate-General for Environment et al., 2017, Eionet 2022). The Danish Baltic coastal meadow is currently considered stable; however, the Atlantic coastal salt marsh is already in regression (EIONET 2022).

Coastal wetlands in Denmark are estimated to have been halved since the middle of the 19th century (Christensen 2017). This loss is primarily due to drainage, cultivation, and containment (Christensen 2017). In drained areas, the tidal impacts and marine floods are partly or totally physically restricted by dikes, sluices or pumps, hence the coastal wetlands habitats gradually degrade and become a freshwater habitat. Furthermore, coastal wetland cannot be considered as isolated habitats since they depend on coastal dynamics and surrounding landscapes. For instance the sediment and drift vegetation deposition from surrounding areas, affect the retrieval or maintenance of coastal wetlands, hence being dependent on changes on surrounding habitats and water dynamics. At present, coastal wetland demarcated under §3 amount to ~467 km^2^ (~1% of the Danish territory; Danish environmental protection agency, 2021). In a sea level rise scenario with rates of 10 mm y^−1^, coastal wetland have two main mechanisms to adapt (Farron et al. 2020, Ebbensgaard et al. 2022): *vertical accretion* (the capacity of accumulating sediment and organic material); and *vertical migration* (inland migration, development of the habitats at higher elevation into land). Vertical accretion is limited to specific coastal wetland where accretion keeps pace with mean sea level rise (MSLR), and coastal squeeze structures like dikes hinders the vertical migration of coastal wetlands.

Local hydrodynamics and the characteristics of sediment and surrounding vegetation define the coastal wetland (salt marsh) dynamic (Bartholdy et al. 2004). The rate of sediment accretion has been positively correlated with area availability and distance from suspended sediment sources (Allen 1990, Bartholdy et al. 2010). However, there is no consensus on to whether this accretion process can cope with an accelerated rate of sea level rise and, if so, for how long (French 2006, Bartholdy et al. 2010). Accretion rates for salt marshes and coastal wetlands have been suggested to range between 2 and 13 mm yr^−1^ (Hatton et al. 1983, Hughes & Paramor 2004, Bartholdy et al. 2010). A more recent study by Horton et al. 2018, estimates that salt marshes are nine times more likely to retreat than expand at MSLR rates ≥7.1 mm yr^−1^. RCP 8.5 projection indicate an average sea level rise of 0.52–0.98 m by 2100 is with a rate during 2081–2100 of 8–16 mm yr^−1^ (IPCC 2021). However, Kirwan et al. 2016 and Hill and Anisfeld 2015 suggest that flooding associated with relative MSLR stimulates the growth of some types of marsh vegetation, enhancing accretion rates more quickly under faster rates of MSLR. The effect of this phenomenon might vary depending on the tidal variation and vertical extent of the marsh area. Furthermore, Simas et al. (2001) and Ganju et al. (2017), found that coastal wetlands with large tidal variation (≥2 m tidal range) are less vulnerable to MSLR as these areas have greater sediment transport hence greater potential for accretion. Tidal range is not the only factor affecting accretion. Strong wind events often increase erosion and resuspension in shallow marine areas, thereby moving sandy material together with organic wrack into the coastal wetlands (French & Spencer 1993, Bartholdy & Aagaard 2000).

The other expansion mechanism is vertical migration (including both shoreline retrogression and lateral expansion). Here an important threat is coastal squeeze. Coastal squeeze is defined as the process where intertidal habitats, like salt marshes and mudflats, are progressively lost or degraded due to the combination of rising sea levels and the presence of fixed man-made coastal defenses (Doody 2004). Construction of climate adaptation structures such as dikes, seawalls, embankments and other coastal infrastructure limits the natural inland migration and may squeeze more exposed coastal habitats like coastal wetlands. This restriction causes these ecosystems to be “squeezed” between rising water levels and static human developments, leading to a reduction in habitat area, biodiversity loss, and diminished ecosystem services (Doody 2004). Historically, damming, land reclamation and drainage to protect agricultural production have been extensive in Denmark. Cities, roads, and other paved infrastructure near the coast also squeeze coastal habitats by pre-occupying future migration space.

A range of models with diverse complexity has been used to estimate the future fate of coastal wetlands, salt marshes and mangrove forest in the future climate. Some of the simpler models include DEM models (elevation and slope) combined with estimated sea level rises and land uses (Molino et al. 2021). The more complex models include, in addition, other parameters from DEM models, such as hydro-geomorphological surrogates. These includes soil typology mapping combined with tidal range, accretion rates or quaternary geology (Borchert et al. 2018, Doughty et al. 2018, Hughes et al. 2022)

Although the present study uses a relatively simple method to estimate the fate of coastal wetlands in Denmark, it does it at national scale, providing a general overview and dimensionality to the effects of Sea-level rises in key coastal habitats. This study is a part of a larger project which examined the impacts of climate related sea level rise (SLR) (both MSLR rise by 2070 and 2120 and 10SS by 2021 MSLR) in a wide range of coastal habitats, at national scale (Ebbensgaard et al. 2022). The overall study examines the effect of SLRs in all terrestrial habitats, including cities, agricultural areas, and freshwater lakes (Ebbensgaard et al. 2022). Ebbensgaard et al. 2022 identifies coastal wetlands as the habitat that will be most affected by both permanent flooding (MSLR) and 10 years storm-surge flooding by 2070 and 2021. In this article, we focus on the effects of permanent flooding by MSLR in coastal nature, primarily on coastal wetlands. The aim of this study is therefore to estimate the order of magnitude of the habitat losses in a climate perspective at national scale.

## Methods and Data

**Fig. 1:**
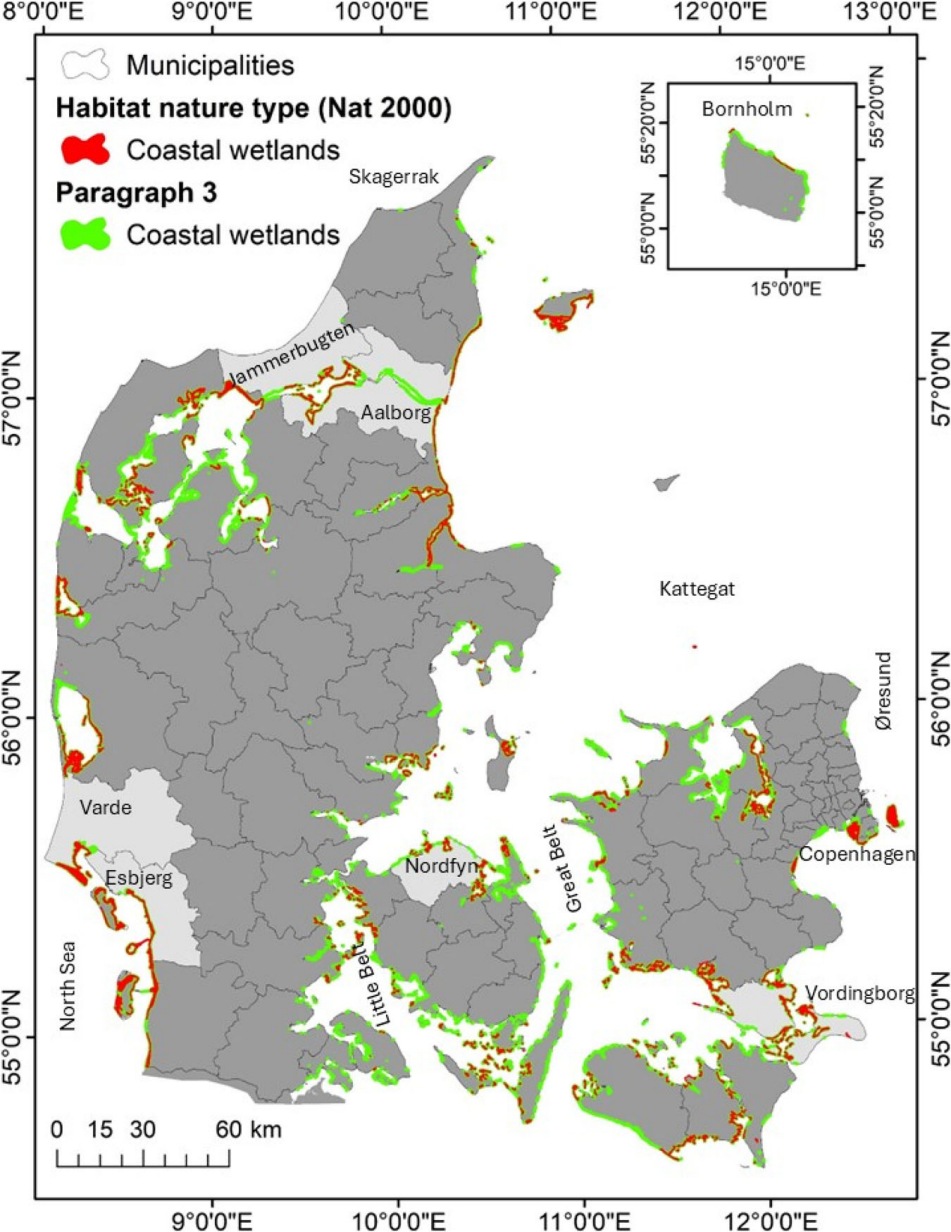
Distribution of §3 and Natura 2000 coastal wetlands in Denmark, 2020. The six selected pilot Danish municipalities are highlighted in light gray

Our study covers all Danish municipalities (98) and focuses on the 76 coastal municipalities that are expected to be affected by future MSLR and 10SS (Fig. 1). We take the 0 m elevation level reference from the Danish vertical reference at 1990 (DVR90), which is used as a national reference. Denmark has a microtidal range of about 0,4 m in the Inner Danish Waters (Øresund, Great Belt, and Little Belt), and about 0,8 m in the west coast (Fig. 1). However, for the present study we consider present and future sea levels at mean sea level. The projection used through all layers of this project was ETRS 1989 UTM Zone 32 N.

The study considered three flooding scenarios: mean sea level rise (MSLR) in 2070 and 2120, and 1 in 10-year storm surge (10 SS) over the MSLR by 2120. The climate scenario used to project the MSLR was RCP 8.5 median (AR5, IPCC 2013). The RCP 8.5 scenario begins with the premise that society will not make coordinated efforts to cut greenhouse gas emissions, hence CO_2_ emissions will continue to grow at the current rate. Floding scenarios were then calculated, following the projection given by the Coupled Model Intercomparison Project (CMIP5) model ensemble, for scenario RCP 8.5 median. For Denmark, this mean around 0.4, 1 and 2 m sea level rise for the MSLR at 2070, 2120 and 10SS at 2021 respectively. However, different geographical areas of Denmark are still rising or sinking compared to the DVR90 reference level, due to the ice recession from the last ice age. Therefore, the exact SLR for each of the run scenarios need to be calibrated for each municipality depending on its geographical location. This calibration is described in detail in the COWI (2017) report “Urban challenges with MSLR and 10SS – COWI for Realdania 2017”. Since all scenarios are done considering mean tidal sea level, the flooded areas estimated by MSLR in 2070 and 2120 are expected to be permanently flooded at high and mean tide (0 to −0,4 m DVR90). On the contrary the calculated areas by 10SS in 2120 represent areas with non-permanent flooding.

The extent of flooding at each municipality was calculated using SCALGO live (SCALGO 2024) for the calibrated MSLR estimated for 2070 and 2120 and 10SS for 2120. SCALGO live is a screening tool widely used in Scandinavia. It is based on a static model (bathtub model) that estimates terrestrial flooding based on a high-definition digital terrain model (DTM/2015 version) and the projected sea level for the chosen scenarios. The Danish DTM is developed and maintained by the Danish Agency for Data Supply and Efficiency (SDFE). DHM/2015 is a high-resolution, lidar-based digital elevation model (DEM) that provides detailed topographical data across Denmark.

In SCALGO Live model, there is a no time dimension, meaning that the ground surface located below the projected water level, it will be inundated following the existing slopes, as long as is connected with the relevant water body (in our case, the sea). With such model, only permanently flooded areas are calculated correctly, hence although we present all data, we have a stronger focus on changes in MSLR, which assume a permanent flooded area. When using a glass plate model to calculate events that are restricted to a certain period of time, ex. storm surges, the model will generally overestimate the flooding, since it does not consider the velocity of the water running through the existing terrain, nor the duration of the weather event. The smaller the inland area subjected to flooding, the more accurate the storm surge will be stimulated. But when there is large extension of areas where the coastal terrain is lower than the simulated storm surge level, the model will overestimate the flooded area, since normally storm surge event last hours to days, while the flooding of large areas can take days to weeks. The Danish DTM used in SCALGO, has a 0,4 m horizontal grid size and cover the entire country. The Danish DTM consists of multiple datasets obtained through airborne laser scanning of the entire country. The emitted laser beam reflects off the terrain or surface, and the time it takes for the reflected signal to return to the aircraft is used to calculate the height of the terrain or surface. The DTM includes infrastructure which modify the natural terrain, such as buildings, but also coastal protection structures. Our study presumed that only all currently existing coastal protection structures (dikes, pumping stations, etc. defined in the existing DTM) would be present in 2070 and 2120, and that no new structures would be put into place or removed, since it is difficult to predict how the country would prioritize the protection of human value vs. nature.

We investigated the fate of coastal Danish territory, using GIS data covering all relevant terrestrial data such as urban demarcations, agricultural land, forestry, protected areas, nature habitats, and the presence and distribution of key species (vegetation and fauna). We focused our analyses on arial data validated by the Danish agency for data supply and infrastructure, the Danish Environmental Protection Agency, and the Danish municipalities (Ebbensgaard et al. 2022).

**Fig. 2:**
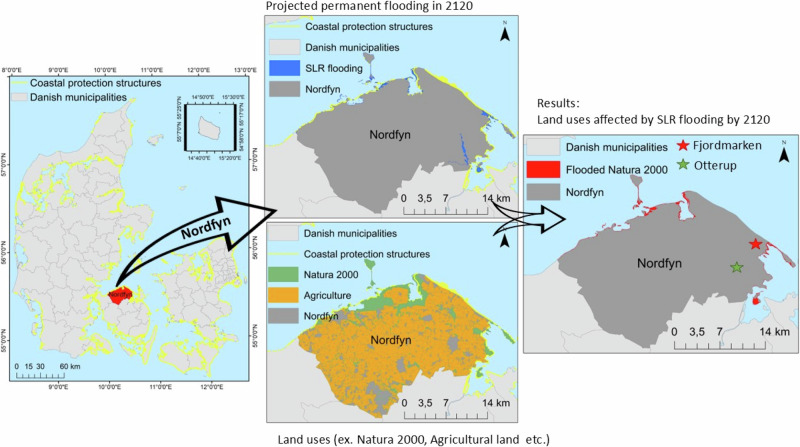
Methodology conceptual diagram. Estimated flooding scenarios here represented by 2120 were calculated using SCALGO live flooding model. The estimated flooding was overlaid on different land uses, hereby represented by Natura 2000 distribution. The resulting map shows land uses which will be flooded by 2120. *Coastal protection structures consist of diverse human infrastructure such as dikes, locks, pumping stations etc

We computed flooded areas for all land uses separately at each municipality. For that, all GIS analyses were performed using ArGIS 10.8. The overall national analysis was performed by using the splitting function. on the GIS raw data layers, using the estimated flooding layer. For example, to estimate the extent of flooding along the different GIS raw layers (cities, coastal habitats, etc.), we split the layers, e.g., the ”protected nature layer from Odense municipality“ with the GIS water level layer (flooding GIS layer) generated by the SCALGO Live software, in this example, ”flooding area in Odense municipality, estimated by MSLR in 2120”. In this way, we obtained the total area affected by flooding in each municipality under our mean MSLR scenarios in 2070 and 2120 (Fig. 2).

We also estimate the area of existing coastal wetlands behind coastal protection structures (e.g., dikes pumping stations etc.). We performed this analysis by extracting all coastal wetlands polygons from the §3 protection layer and splitting them with the National mapped coastal protection structures (lines). We deleted areas outside the coastal protection lines (towards the sea) and summed the areas of the remaining polygons.

For six selected Danish municipalities, we performed additional analyses. Here we analyzed the distribution of coastal wetlands in relation to the Danish terrain at present and as predicted for 2120, given MSLR expectations under scenario RCP 8.5. The DTM was extracted from SCALGO live. We show the areal distribution of coastal wetlands at present and estimated the potential for future development in 2120. To estimate the distribution of coastal wetlands in 2120, we assumed a new coastline based on the estimated future mean sea level. We further assume the following: only coastal areas with existing coastal meadow will be able to vertically migrate; coastal wetlands that are currently laying behind dikes will not be affected by MSLRs nor 10SS (as long as the height of the dike exceeds the MSLR or 10SS); existing dikes will remain at their current heights and the new coastal wetlands will be situated at the same elevation as the present coastal wetlands are placed today, in relation with the future mean sea level (~1 m higher, corrected for the sea level increase, due to crustal motion, for each municipality as explained above). Our rough estimate of future coastal wetlands includes wetlands that would emerge on land currently used for other purposes, such as agriculture, cities, or other protected nature habitats. Some of the estimated future coastal wetlands are not expected to be realized, as some landowners most likely would prefer to drain or dike in their land, unless other arrangements take place. Thus, our estimate can be considered the best-case scenario for coastal wetlands.

Finally, as an example, we performed more detailed analyses in a specific lowland area of Denmark, Nordfyn municipality. In this detailed study, we estimated the potential distribution of future coastal wetlands in Fjordmarken, Nordfyn municipality (Fig. 2), Denmark under two scenarios. In scenario 1 we did not modify any existing coastal protection structures, in scenario 2, we removed all coastal protection structures. With this example, we aimed to assess the impact of very different climate adaptation strategies, where the water is invited in or kept out by coastal reinforcement structures (dikes). In both scenarios, we imposed the average water levels in 2120 projected under RCP 8.5. We then calculated and compared the extent of future coastal wetlands under the two protection regimes, following the procedures explained above.

## Results

### National Analyses

Our study shows that 76 out of Denmark’s 98 municipalities will be affected by permanent marine flooding by 2070 and that continued MSLR would affect the same municipalities, but to a greater extent, by 2120. We estimate a permanent flooded area of ~18,100 ha by 2070, expanding to 72,700 ha by 2120. From this, we quantified the loss of protected natural habitats: ~10,000 ha (3%) of the Danish Natura 2000 areas would be permanently flooded by 2070; 36,000 ha (11%) by 2120 (Table 1). By far, the most-affected habitats are open coastal habitats including dunes, heathland, freshwater meadows, dry grassland, and coastal wetlands. ~6,000 and 21,000 ha (3.8 and 13.7%) of these areas under the Natura 2000 regulation are expected to be flooded by 2070 and 2120, respectively. Furthermore, ~10,000 and 33,000 ha (2.7 and 8.9%) of open land habitats under §3 nature regulation will be flooded by 2070 and 2120, respectively (Table 1). At the same time, we find relatively low losses in cities, forests, and agricultural land, where we expect a maximal loss of 0.6, 0.3 and 1.6% respectively by 2120. However, even if the relative impact to agricultural is low, 0.3 and 1.6% lost by 2070 and 2120, the area lost is not negligible: ~8000 and 37,000 ha by 2070 and 2120 (Table 1).

**Table 1:** MSLR and 10SS flooding in Danish coastal areas (98 municipalities)

	Total area	Permanently flooded (MSLR)				Storm- flooded area (10 SS)	
	ha	ha		%		ha	%
		2070	2120	2070	2120	2120	2120
**Agricultural land**	2,292,678	7732	36,897	0.3	1.6	191,251	8.3
**Forest**	508,603	727	1551	0.1	0.3	16,398	27.4
**Cities**	272,087	500	1621	0.2	0.6	22,429	8.3
**§3 nature, open-coastal habitats**^a^	366,899	9855	32,551	2.7	8.9	49,476	32.4
**Natura 2000**	330,952	10,010	36,005	3	10.9	110,665	33.4
**Natura 2000, open-coastal habitats**^a^	152,584	5812	20,846	3.8	13.7	49,478	33,4

^a^Open-coastal habitat types (lakes, dune, heathland, coastal meadow, freshwater meadow, dry grassland, and marsh/bog)

Permanently flooded area in diverse land uses by projected increased average sea levels in 2070 and 2120 Storm flooded area in diverse land uses by estimated peak water level under 1 in 10-year storm surge in 2120

During 1 in 10 years storm surge events by 2120 at MSLR, it is estimated that up to 191,000 ha (8,3%) of agricultural land might be affected by non-permanent flooding. Up to ~16,000 of forest and ~110,000 Ha (17 and 33%) of forest and Natura 2000 will be affected by non-permanent frequent flooding.

Looking further into the open coastal habitats under §3 nature protection (see Table 2), coastal wetlands will suffer the highest losses: ~6300 and 20,000 ha (14.3 and 44.7%) by 2070 and 2120 respectively. The impacted areas of lakes, freshwater meadows and marshes/bogs are predicted to be ~1500 and 5200 ha (3 and 9.9%), 1000 and 4000 ha (1.3 and 4.5%) and ~650 and 3078 ha (0.8 and 3.8%), respectively, by 2070 and 2120. The current distribution of §3 coastal wetlands, which now cover ~44,500 ha in Denmark, can be seen in Fig. 1. Regarding the flooding expected by 10SS, since it is not permanent, the different habitats will be affected differently. The greatest extent of non-permanent flooding is associated with coastal wetlands, with ~40,000 ha, followed by freshwater meadows and lakes with ~18,000 ha.

**Table 2:** Impacts of MSLR and 10SS flooding in Danish §3, open-land habitats

	Total area	Permanently flooded area (MSLR)				Storm- flooded area (10 SS)	
	ha	ha		%		ha	%
		2070	2120	2070	2120	2120	2120
**Freshwater meadow**	85,592	1,102	3,812	1.3	4.5	18,214	21.3
**Heath**	72,242	92	311	0.1	0.4	6,115	8.5
**Marsh/bog**	80,462	651	3,078	0.8	3.8	15,263	19
**Dry grassland**	31,415	89	242	0.3	0.8	2,239	7.1
**Lakes**	52,697	1,575	5,201	3	9.9	18,802	35.7
**Coastal wetland**	44,491	6,346	19,908	14.3	44.7	40,080	90.1
*Total*	*366,899*	*9,855*	*32,551*	*2.7*	*8.9*	*100,712*	*27.4*

Permanently flooded area in all open-land habitats under §3 regulation by projected increased average sea levels in 2070 and 2120. Storm-flooded area in in all open-land habitats under §3 regulation by estimated peak water levels under 1 in 10-year storm surge in 2120

Further, we investigated how current coastal wetlands under §3 are distributed in relation to the already existing coastal protection structures (overview map in Fig. 1). Our results show that already now, large areas of the country have been and are being artificially drained and/or protected by dikes. About 14% of the existing §3 coastal wetlands lay behind by dikes and/or have been drained (via pumping stations).

### In-depth Analysis of Pilot Municipalities

Six municipalities were selected to represent the geographical and geophysical variety of Danish coastal exposure and tidal range. These 6 pilot municipalities are: Jammerbugt, Aalborg, Esbjerg, Varde, Nordfyn and Vordingborg (Fig. 1).

**Fig. 3:**
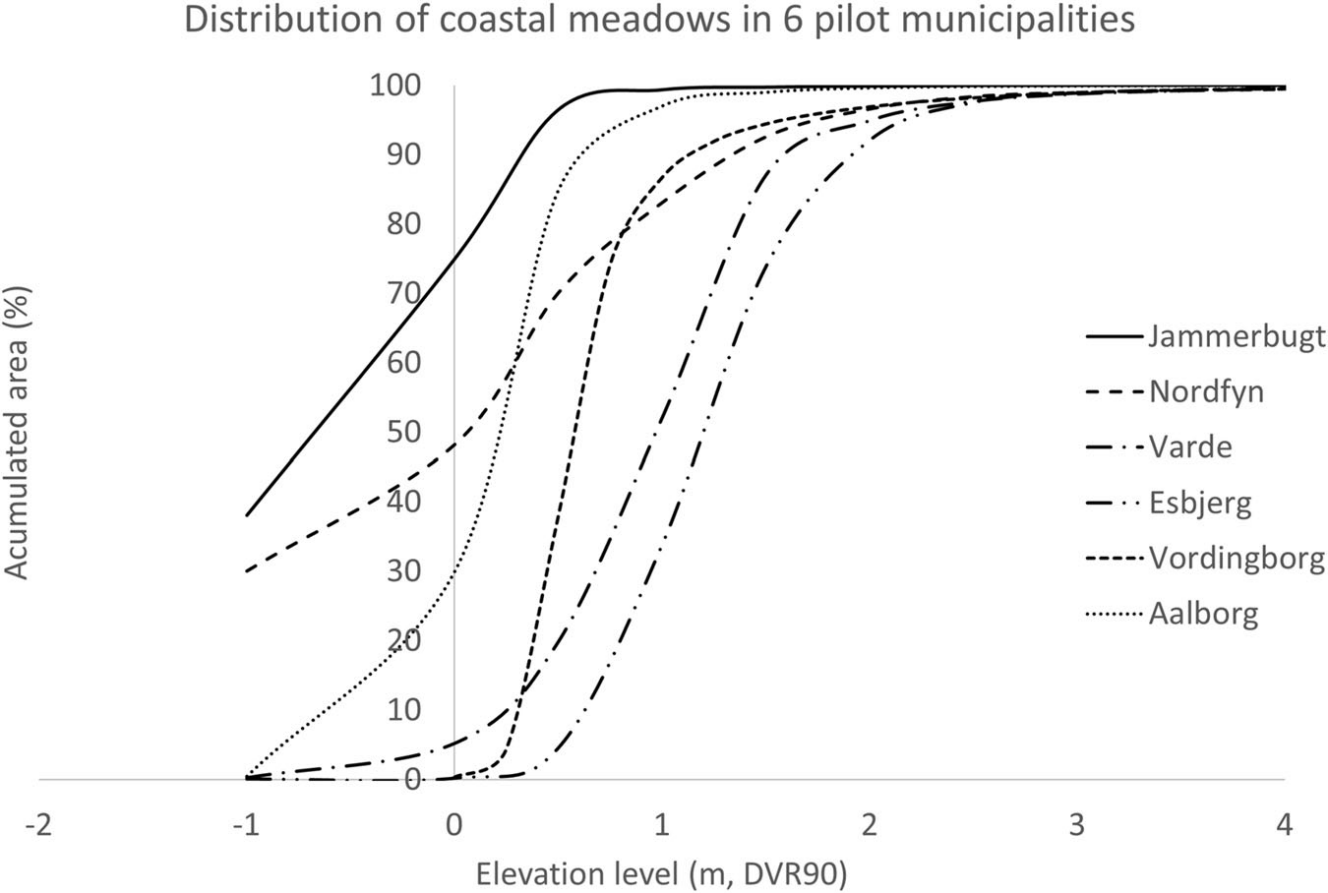
Vertical distribution of coastal wetlands in 6 Danish municipalities. Elevation reference Danish Vertical Reference 1990 (DVR90)

Here we investigated in detail the vertical distribution of the present coastal wetlands. Figure 3 illustrates three tendencies. Nordfyn and Jammerbugt have relatively large areas at or below the current mean sea level datum (0.0 m DVR90), 48% and around 75% respectively. These two municipalities have also a high abundance of dikes and pumping stations. In Vordingborg and Aalborg, most coastal wetlands 87% and 97% respectively, are 1 m or less above the DVR90 datum. Finally, Esbjerg and Varde show coastal wetlands with a wider terrain vertical distribution (inland), in which about 34% and 52% respectively, of their coastal wetlands are located 1 m above the current mean sea level (Fig. 3).

**Fig. 4:**
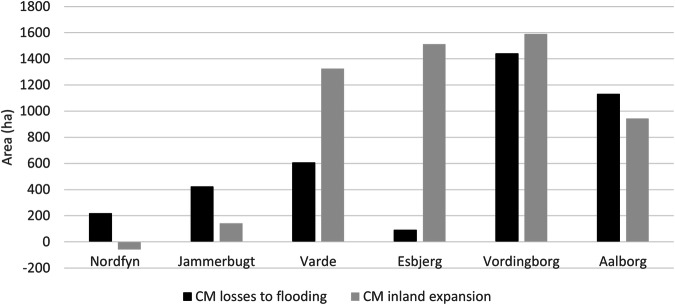
Projected change of coastal wetlands (CM) in 6 municipalities in 2120, after average MSLRs

Our analyses indicates that by 2120, Nordfyn, Jammerbugt and Aalborg municipalities will experience a loss of coastal meadow area compared to present conditions (Fig. 4). Vordingborg will gain small areas, and topographic conditions in Varde and Esbjerg municipalities might lead to a large increase (Table 3).

**Table 3:** Area of different land uses under predicted potential coastal wetlands by 2120 in our pilot municipalities

	Area	
	ha	%
**Agricultural land**	6767	2
**Natura 2000, open-land habitats**^**a**^	4901	16
**3 nature, open-land habitats**^**a**^	5570	8.2
**Natura 200o lakes**	161	7.5
**Freshwater breeding areas**^**b**^	36	25.1

^**a**^Open-land habitat types (lakes, dune, heathland, freshwater meadow, dry grassland, and marsh/bog)

^b^Freshwater breeding grounds are mapped habitats of special importance for protected amphibian species

**Fig. 5:**
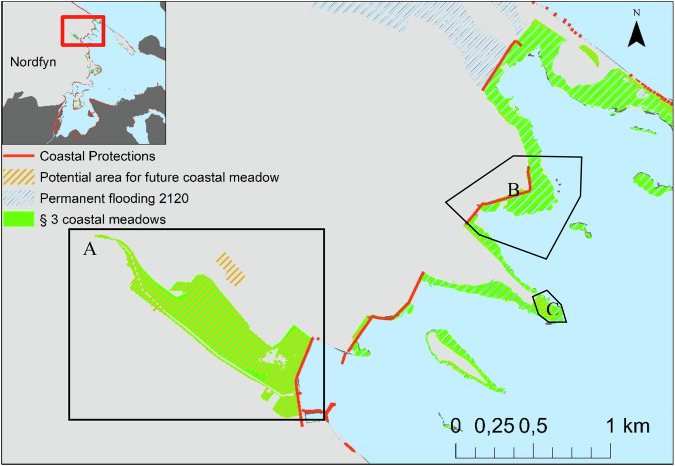
Present and predicted 2120 distribution of coastal wetlands in Nordfyn, Denmark. The selected area **A** is Fjordmarken, a drained area reclaimed from the sea more than 150 years ago. Area **B** will be in coastal squeeze by 2120. **C** is an area with potential for vertical migration for coastal wetlands by 2120

Large areas of the current coastal wetlands will be coastal squeezed by the current coastal protection structures. Figure 5 visualizes three ways in which Nordfyn’s coastal meadow distribution will change by 2120. First, a large area of coastal meadow, currently behind a dike, will remain (Fig. 5A). Second most of the coastal meadow areas in front of dikes will disappear due to coastal squeezing (Fig. 5B). Finally, coastal meadow areas might expand vertically, whereas others will not, due to the terrain distribution of the future coast (Fig. 5C).

We estimate that by 2120, ~7000 ha of current agricultural land will be conducive to coastal marshes. These areas only account for 2% of the total existing agricultural land in the pilot municipalities. The next-largest potential coastal marsh environments are currently open-coastal habitats, where ~5000 and 5600 ha (16 and 8.2%), are under Natura 2000 and §3 respectively. This relative loss might be an underestimated, since in this calculation we do not consider that other open-coastal nature habitats, e.g., moors, heaths etc. also will migrate vertically (inland) to the closest existing barrier (dike, city, agricultural land etc.). The potential for transformation of current freshwater habitats into coastal wetlands is smaller, 161 ha for lakes and 36 ha for freshwater breeding grounds. However, the relative loss might be of importance, 7.5 and 25.1%. Notice as well that this study does not consider projections of climate-induced freshwater flooding by 2120.

**Fig. 6:**
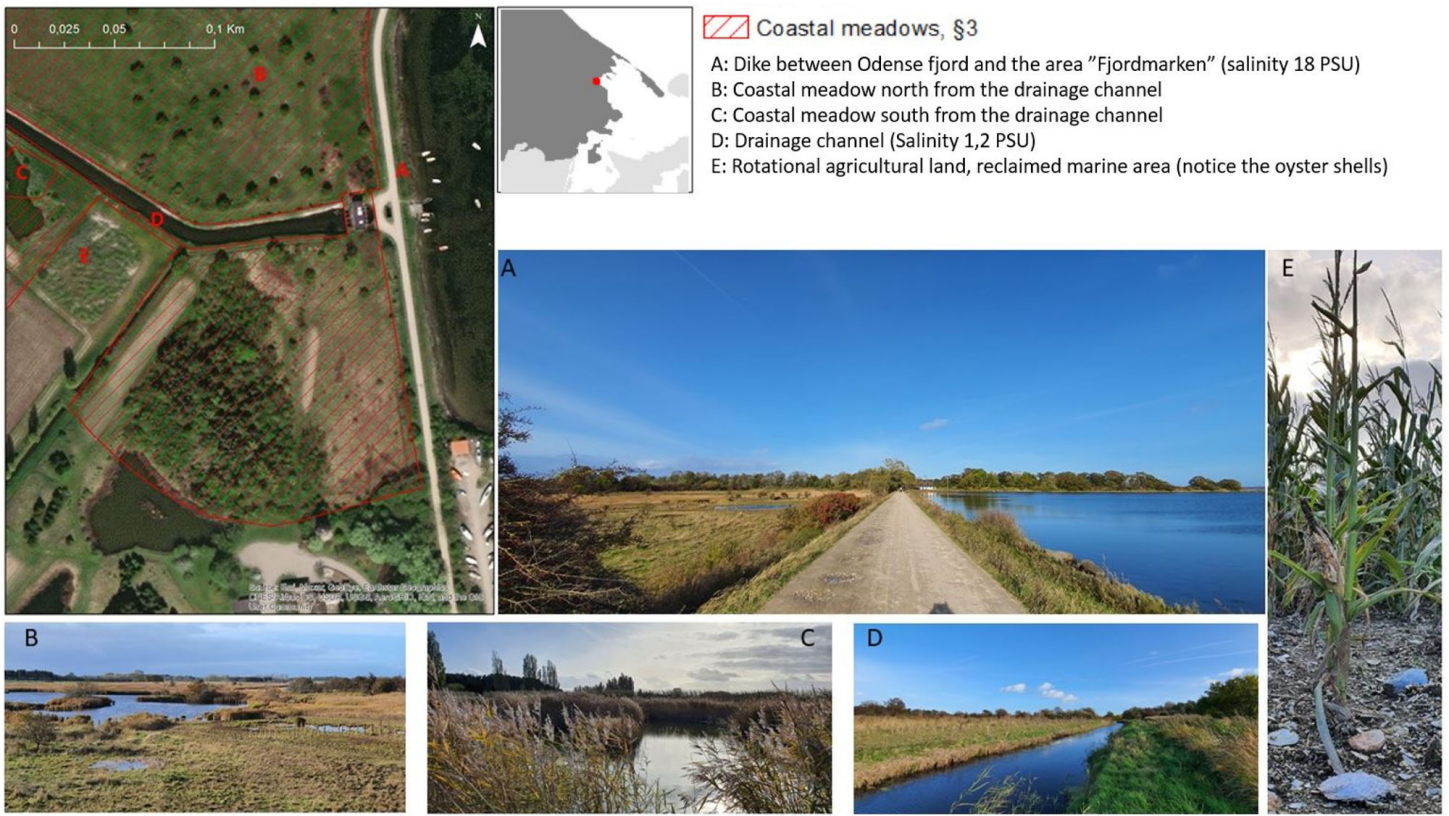
Existing coastal wetlands (coastal meadow) at Fjordmarken, Nordfyn, Denmark. (Photos by Paula Canal-Vergés). **A** Dike between Odense fjord and the area of "Fjordmarken". **B** Coastal meadows north from the drained channel. **C** Coastal meadow south from the drainage channel. **D** Drainage channel. **E** Rotational agricultural land, reclaimed from the sea.

Fjordmarken is a drained area, highly influenced by freshwater, situated in the NW part of Odense fjord (Fig. 6). The area, previously seabed (Fig. 6E), was drained in 1815 (Basse 1957). Most of the drained area is dedicated to agriculture, but a relatively large area was bought by Funen County in 1996 and handed over in 2007 to the Danish Nature Agency, which turned it into a protected nature area (under both Natura 2000 and §3, Fig. 6B, C). The area is located in the northeast of Otterup, Nordfyn municipality, Denmark (Fig. 2). The salinity at the drainage channel (hence around the mapped coastal meadow) was very low, mean 1.2 PSU (freshwater, measured by the author, Fig. 6D), whereas the salinity on the other side of the dike was mean 18 PSU (measured by the author, Fig. 6A), characteristic of many Danish fjords (Fig. 6).

**Fig. 7:**
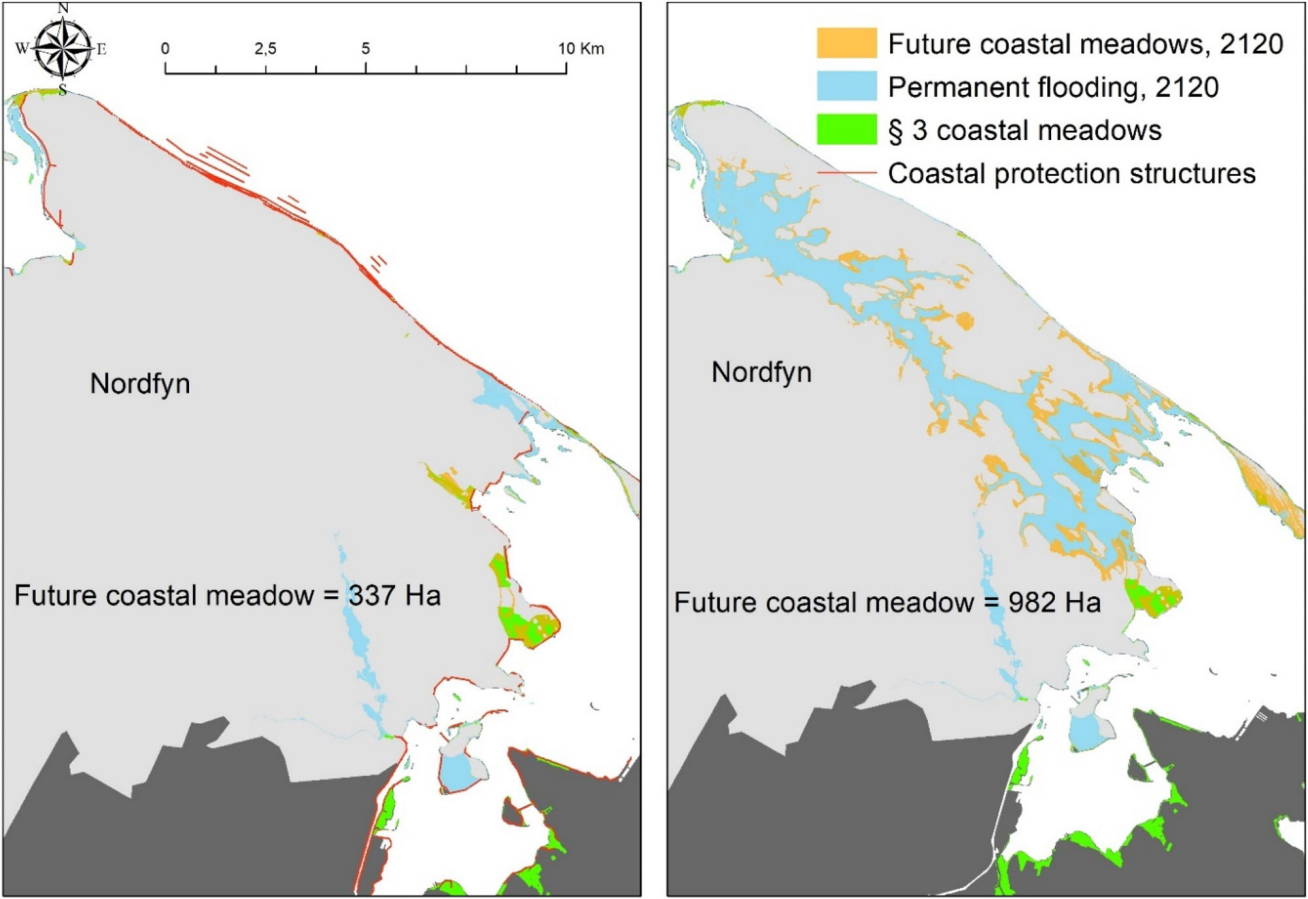
Predicted future coastal wetlands (coastal meadows) in Nordfyn municipality after sea level rising by 2120, with and without the removal of coastal protection structures

We use Fjordmarken as an example of the potential consequences of choosing to remove or increase coastal protection. Here, we investigated more in detail the effects of removing coastal protection structures under 2120 MSLR conditions, as example for potential future solutions to restore coastal wetlands in reclaimed areas (Fig. 7). We recalculated the potential areas for future coastal wetlands and compared them to those with dikes. Here we find that the removal of coastal protection structures around Fjordmarken might increase the potential for coastal wetlands development from 337 ha to 982 ha in 2120 (Fig. 7). However, the landscape consequences are extreme, with a new island system appearing in the north of Funen (Fig. 7). The flooded area increased from 598 to 4309 ha with and without Fjordmarken’s dikes and pumps. The increased flooded area would also force the losses of ~1500 ha of agricultural land, ~280 ha of open-land habitats and ~60 ha of forest. A few areas of rural houses would be impacted as well.

## Discussion

There are more complex and accurate methods to estimate the geographical areas that will be subjected to marine flooding due to mean sea level rise and extreme weather events such as those described in (Raposa et al. 2016, Borchert et al. 2018, Doughty et al. 2018, Hughes et al. 2022). However, most of the Danish coastal wetlands are located in their inner waters with a microtidal range of ~0,4 m, which reduces the error introduced by not including wave models, or tidal ranges in our calculations. Our selected method gives therefore a rough estimate; however, we have done the analyses at a national level. There is also a wide range of future RPC scenarios that could be chosen to estimate the fate of coastal habitats. We selected both our RPC 8.5 as our scenario, although it is the most extreme of them and assume that not mayor improvements/changes will take place. Our scenarios selection as well as the methods used, follows the general guidelines and tools used in the Danish climate adaptation plans (Nordic council of ministers 2023). Our overall aim was to investigate whether, the current plans were considering coastal nature as part of the climate adaptation and if so to what extent. Furthermore, our study aimed to estimate specifically, the order of magnitude for the potential losses of coastal wetlands due to permanent flooding caused by MSLR. We further estimated areas where the land will be flooded on average once in ten years (10 SS), following again the Danish adaptation plans.

Denmark is a low land, with a large area of the country occupied by human uses such as agriculture and urbanization. Historically, it is estimated that around 40,000 ha of the Danish territory were taken from the sea, mostly for agricultural exploitation already by 1986 (Waagepetersen et al. 1986). Furthermore, our study shows that, only ~0.3% of the urban areas will be affected by permanent flooding due to MSLR, compared to ~13% of Natura 2000 sites (Table 1). This illustrates that the Danish spatial plans have over the past decade been reinforcing the coast to protect human infrastructure without considering existing and future coastal wetland development. These coastal protection structures reinforce the coastal squeeze on natural habitats, especially coastal wetlands. Not all municipalities have finished implementing these plans, which are intended to engage with the ocean environment that is expected in 100 years. The plans envision more dikes and drained areas, increasing the coastal squeeze on natural habitats. Therefore, unless we see a radical change in government priorities, or policies, the biodiversity of Danish coastal habitats are not expected to improve over time. Fortunately, change is possible. In the USA, Borchert et al. 2018, finds that the potential for coastal squeeze is highest in estuaries containing major metropolitan areas that extend into low lying lands. This is the case for most Danish municipalities, which locate their main cities towards the sea. In England, coastal squeeze was identified as a major threat for coastal meadows and salt marshes almost 40 years ago (Doody 2004). Twenty years ago, the English authorities started a process to try reversing the accumulated negative effects of coastal squeeze and land reclamation (Doody 2004). These changes had the effect of an engineering solution that reinforced coastal habitat protection. For instance, they used solutions as managed retreat (allowing certain low risk areas to erode and flooded), soft engineering (beach nourishing, dune restoration etc.), living shorelines (combination of natural elements such as marsh or oyster reefs with engineered structures), and hybrid defenses (combining hard defenses as dikes with soft ones such as dunes etc.) (Doody 2004). This choice was reinforced more by declining agricultural interest in (and returns from) coastal areas (Doody 2004). A similar shift in political priorities might underly a similar policy change in Denmark. That is, biodiversity-supporting outcomes can be realized without a strong environmental movement. At present, relatively small changes to include nature are taking place in some of the Danish policies. An example is the flooding directive. In Denmark, the Danish Coastal Authority (DCA), is responsible for implementing EU Floods Directive. At present, DCA is reviewing the preliminary flood risk assessment (PFRA) for the third cycle of the directive (2022–2027). In the new PFRA, DCA is analysing the consequences of flooding in three categories: economic; social; and for the first time, nature & environment (personal communication with the DCA 2023). These analyses are made for coastal and fluvial flooding. The new model for nature and environment is a rough first model, due to time and data constrains, but includes consequences on different ecosystems as well as loss of recreational value. The DCA’s plan is that the new national environment flood risk assessment together with the new national economic and social flood risk assessments will be used to identify the areas with potential significant flood risk (Andersen 2022).

In Denmark, a large area of agricultural land will be at risk of flooding as the sea level rises during the coming 50 to 100 years (up to 36,900 ha, Table 1). It is uncertain what will happen in these areas. The answer depends highly on the economic model the country decides to follow. There would be a net loss of productive agricultural land, so maintaining this industry at its present size would involve re-purposing land that currently is used for other purposes. It is estimated that at present, 54% of the Danish territory is dedicated to agriculture, 14% to urbanization, 13% to forest (some of which are also agriculture), 9% nature, 2.7% rivers and lakes and 1.6% is not classified (Denmark Statistic 2021). The space to expand agriculture in other areas inland in a future scenario, is therefore very limited. If agricultural production remains a priority and should keep the current area extension or increase production, additional dams, dikes and reclamations can be expected and, with them, intensified threats to key habitats. If we consider that about 11% of the existing Nature (Natura 2000 areas) might be lost due to average MSLR by 2120, and only 1.3% of the agricultural land will be affected, the situation will only worsen.

Our results show that of all open-coastal habitats, Danish coastal wetlands will suffer the highest losses at MSLR: we estimate that about 14% of the existing coastal wetlands will be permanently flooded by 2070, and 45% by 2120 (Table 2). This national loss is of even more importance, because Denmark contains 78.5% of Europe’s coastal meadows in the continental zone and 14.5% in the Atlantic zone (EIONET 2022). Horton et al. 2018 found equally alarming results in their study, where they estimated with 80% likelihood that all English salt marshes would retreat due to MSLR by 2100, with areas in southern and eastern England in retreat by 2040. Molino et al. (2021), find that, for Chesapeake Bay, coastal marsh expansion due to SLR will only displace coastal forest given the availability of the right migration paths, and otherwise will end in a net reduction of the marshes due to coastal erosion and lack of accretion. For the Danish meadows, the transitioning will be towards low land vegetation such as freshwater wetlands, heath bog or lakes, with the forest on higher lands. However, the same migration path mechanisms found in Borchert et al. 2018 or Molino et al. 2021 are applicable, in our study. Hence, we expect a net loss if the migration paths are not available and suitable. Furthermore, some of the freshwater habitats, such as lakes and freshwater wetlands might be at risk due to 10SS, which will induce marine flooding. It is uncertain whether the frequency of these events will suffice to transition of these habitats into coastal wetlands, or rather transition of these to less halophile habitats.

We estimate that ~14% of the existing coastal wetlands are already behind dikes or other coastal protection structures. In our study, areas subjected to high damming, like Nordfyn or Jammerbugt municipalities, coincide with a distribution of coastal wetlands in which a high percentage lays below current sea level. Coastal wetlands in Jammerbugt and Nordfyn are narrow, with most occurring from below current sea level to about below 1 m (Fig. 3). Dammed areas have lost the direct connection with the sea (tidal effect) and become much more impacted by freshwater sources, as seen in our example in Fjordmarken (Fig. 6). In these areas, the accretion/erosion processes typically found in coastal marshes is permanently changed. Furthermore, this study likely underestimates the desalination of these habitats since climate induced freshwater flooding is not considered. The Sixth Assessment Repot (AR6) of the IPCC (IPCC. 2021), highlighted the increment of overall winter precipitation as well as the increase of extreme rain events, which will increase the overall freshwater hydraulic pressure to these very low areas. Despite the freshwater influence, our example from Fjordmarken, is categorized as coastal meadow based on a botanical index. However, the vegetation on coastal wetlands consist of halophytes (salt-tolerant plants). Halophytes as well as the associated fauna are adapted to highly saline environments, hence, these habitats will eventually turn into freshwater meadows or other open-coastal habitats (such as dry grassland or other). In fact, according to Christensen 2017, coastal wetlands in Denmark have been halved since the middle of the 19th century. He associates this loss to drainage, cultivation and containment, which coincides with our findings.

Coastal squeeze goes hand in hand with the high degree of damming in Denmark. All dike-protected coastal areas will be coastal-squeezed. In Denmark, coastal wetlands placed in front of dammed areas will be gradually lost up to 2120, since the climate adaptation plans are prepared for average MSLR by 2120 (COWI 2017). Our results show that coastal wetlands have already declined to such an extent that the problem should be understood as one of great importance in Denmark, and one that will increase over time, since counterproductive infrastructure continues to be added in cities, summerhouse areas and elsewhere. Our analyses shows that Nordfyn, Jammerbugt and Aalborg (tidal range 0.4 m) will lose area even if their coastal wetlands are allowed to migrate vertically (inland) (Fig. 4). In Nordfyn and Jammerbugt, coastal squeeze is already caused by damming. In Aalborg paved areas that promote coastal squeeze are very close to the coastal meadow habitats. This situation is more extreme in Nordfyn (Figs. 4–6), where by 2120, coastal squeeze will force the disappearance of all coastal wetlands that are not already placed in dammed areas (hence transitioning to freshwater habitats). Esbjerg and Varde municipalities show a very large potential for increasing their vertical distribution (inland) over time (Fig. 4). Esbjerg will not suffer great coastal wetlands losses, due to the higher coastal terrain elevation. However, the realization of the predicted expansion of its coastal wetlands at MSLR in 2120, due to its location, will be mostly at cost of other low vegetation habitats, and depending as stated of the presence of the right migration paths (Ebbensgaard et al. 2022, Molino et al. 2021). On the contrary, Varde, will suffer some loses due to its lower slope of its coast. However, in Varde there are large areas with agricultural fields at elevations where coastal wetlands they could be transformed to coastal wetlands given projected (Ebbensgaard 2022).Varde and Esbjerg municipalities are located at the Danish west coast, with a tidal range of about 2 m (rather than 0.4 m in the inner Danish waters), which according to Raposa et al. 2016, gives them a slightly higher resilience. Our study is likely underestimating the accretion rates for these areas, hence overestimating the flooded area and therefore the potential for vertical (inland) migration. For these two municipalities, and in general for the west coast, where the tidal range is larger, further local analyses to estimate coastal meadow resilience as those described in Raposa et al. 2016 or Doughty et al. 2018 are needed. Finally, coastal wetlands (tidal range 0.4 m) in Vordingborg show a certain potential for inland expansion by 2120. In our scenarios, we do not assume that agricultural areas and or urban areas will be further reinforced or dammed, which provides our study with character of “best case scenario” compared to the estimated effects of coastal nature. From our study, we can conclude that for the six pilot municipalities, inland expansion of coastal wetlands, if possible, would be mostly at the cost of agricultural land and, to a lower degree, of other open-coastal habitats (Table 3). Furthermore, as can be seen at Fjordmarken (Fig. 7), if coastal protection structures are removed, large areas of agricultural fields will be flooded at present mean sea level. Within our pilot municipalities, a total of ~7,000 ha of agricultural land area situated at terrain elevation that coincide with territory that is conducive for coastal meadow expansion by 2120. These areas will only become available, however, if the landowners actively choose to stop exploitation and allow them to transition into natural habitats. Numerous studies around the world have documented the retreat of farmland and its replacement with salt marshes as the sea level rises (Nicholls & Leatherman 1995, Doody 2004, Clark et al. 2015, Fagherazzi et al. 2019). However, as stated by Doody 2004, and considering the findings of the present study, most of these changes in land use are strongly influenced by the national economic and political priorities.

In areas where coastal wetlands will not be allowed to migrate vertically (inland), the only coping mechanisms that would allow this habitat to survive is coastal accretion. But accretion depends on many local parameters, such as vegetation type and management, flooding time, or proximity to available sediment pools (Horton et al. 2018, Fagherazzi et al. 2019). Bartholdy et al. (2010) found that in the Danish Wadden Sea, the vegetation from coastal wetlands start at an elevation of 0.8 m. Our results from two pilot locations in this study (Varde and Esbjerg) support Bartholy’s findings. Existing meadows in both municipalities are located at higher elevation levels, compared to the rest of the studied meadows; with <10% (Esbjerg) and 18% (Varde) of the total distribution below 0.5 m. Our distribution data support the findings from Simas et al. (2001) and Ganju et al. (2017) which showed that areas with low tidal amplitude might be more vulnerable to MSLR, both due to their estimated accretion but also due to the narrower natural distribution (narrow vertical distribution). Recent studies from Farron et al. (2020) suggest that amount of sediment available is one of the most critical factors for coastal meadows expansion. In U.S. salt marshes with varying tidal differences, Ganju et al. (2017), found that generalized sediment deposition was insufficient for the accretion process to keep pace with MSLR. The present study is an overall national estimate, intended to describe and roughly quantify the existing shortcomings in the climate adaptation plans for the management of natural habitats. We highlight the importance of more detailed studies to improve local plans. For instance, considering the proximity of the studied coastal wetlands to available sediment pools, as well as the local hydrodynamics, will improve our prediction locally. Van Eerdt (1985) found that the plant community and its management influenced the rate of erosion, and Bouma et al. (2005) and Raposa et al. (2016) found that above-ground plant traits can influence the interaction of vegetation and sediment, hence the meadows resilience. For example, a plant’s stiffness and density may affect sedimentation rates (Bouma et al. 2005). In our study, we did not evaluate the species or the management (grazed or mowed vs unmanaged) of the studied coastal wetlands; doing so would again improve local predictions.

Overall, deeper knowledge and good quality data such as land cover habitat mapping as well as the inclusion of other parameters such as soil type, mapping of agricultural drains, ground water levels etc. for the specific local areas, are necessary to improve the accuracy of the predictions for the development of local coastal wetlands development. As well, using more complex models which include, local hydrodynamics, tidal amplitude, accretion rates, hydro-geomorphological surrogates etc. as that described for instance in Raposa et al. (2016), Borchert et al. (2018), Doughty et al. (2018) and Hughes et al. (2022) will improve the accuracy of the prediction. These improvements however will not cover the uncertainties associated with the IPCC SLR projections. Some of the land cover information, such as precise habitat mapping, habitat quality or specific agricultural uses, can be collected efficiently using remote sensing technology on Unmanned Aerial Vehicles (UAV) (Svane et al. 2020). Here, vegetation coverage and species fitness can be estimated using UAV images and refined with the use of multispectral or infrared sensors. In many populated areas, marine debris (algae and eelgrass wrack), which contributes to vertical accretion, is actively removed from shores for the benefit of tourists, while in other areas the wrack is unmanaged hence associated with highly variable retention times (Möller et al., 2021). Retention time, hence permanent accretion, depends on hydrodynamics, the conditions of surrounding benthic habitats and characteristics of the coastline (Raposa et al. 2016, Möller et al., 2021). When the beach debris is removed, the organic material, which would deposit in coastal wetlands during storms, will be lost, decreasing the potential for vertical accretion for these areas (Möller et al., 2021).

A potential method for reestablishing and or maintaining coastal wetlands is to remove dams from reclaimed areas or to allow gradual coastal flooding of e.g., agricultural land. Re-flooding of reclaimed land, like the Fjordmarken scenario proposed in the present study, was done in Vigelsø, Funen Denmark. Vigelsø was used for agricultural purposes for more than 150 years. The storm surge that followed storm Bodil in December 2013 broke through the dikes around the dammed southern part of the island (Naturstyrelsen Fyn 2018, Petersen and Strandgaard, 2021). During the flood, 25 ha of coastal meadow disappeared, and the habitats of the many meadow birds were greatly reduced. Instead of repairing the dike, the Danish Nature Agency chose to open it up further and landscape slightly higher beach meadows by sacrificing the old agricultural land. The aim of this project was therefore to restore coastal wetlands (specifically, coastal meadows). New coastal meadows were established by excavating soil from moraine hills towards the flooded areas and moving the soil out where there was flooding. In total approx. 100,000 m³ of soil and debris was removed, which gave ~27 ha new coastal meadow. The established coastal meadow was grazed and, within 3 years of restoration, evaluated to be in good quality status (according to Natura 2000 criteria). Highly diverse vegetation (characteristic of this habitat) as well as a wide diversity of birds has already established in the new areas (Naturstyrelsen Fyn 2018, Petersen and Strandgaard, 2021).

Our study shows that up to 191,250 ha of coast near agricultural areas will be subjected to frequent flooding (10SS) by 2120 in Denmark. This gives an order of magnitude of the land area with potential for future coastal wetlands, given the land is naturalized, landscaped or bioengineered to frame coastal meadows (as it was on Vigelsø). However, as discussed, neither our analyses nor Ebbensgaard et al. (2022) are sufficiently detailed to point to specific grounds and actions. We instead limit ourselves to identifying general areas for further analyses including local hydrodynamics, description of soils (both within and surrounding the candidate area for restoration), vegetation description of neighboring areas, and so on.

Finally, our study indicates that if the current economic and political/legislative priorities do not change, and human infrastructure and agriculture remains the focus of the climate adaptation plans nature will keep being neglected. In a climate perspective, the Danish coastal nature will keep increasingly deteriorating. Furthermore, the nationally and internationally valuable Danish coastal wetlands will suffer great losses. Our study also shows the general consequences of not including/prioritizing nature conservation nor ecosystem-based adaptation in the climate adaptation plans for cities and municipalities. Hence, we highlight the importance to include nature more thoroughly into the national plans and policies.

## Conclusion

Our study shows that by 2070 and especially by 2120, large areas of coastal habitat will be lost due to average sea level rise (9–14% of current coastal habitat). Of all coastal habitats, coastal wetlands will be the most affected, with an estimated loss of 40% of their current distribution by 2120, due primarily to coastal squeeze. Other land uses such as agriculture, urbanization or forestry will experience much lower relative impacts (0.3 to 1.6%), due to, among other reasons, the government’s prioritization to protect infrastructure in its climate adaptation plans.

Our pilot studies show that coastal wetlands/salt marshes have two distinct distribution patterns. On the west coast, these habitats have a wider distribution, reaching higher elevation, and are subjected to a tidal range of ~2 m. In the rest of Denmark, coastal wetlands have a narrower distribution, rise to lower elevations, and are exposed to a microtidal regime of around 0.4 m. Considering the projected (RCP 8.5 median) sea level rise, and in agreement with Doody (2004), western coastal wetlands are expected to be more resilient (although still under recession) than those located in Denmark’s inland waters.

We found that 14% of existing coastal wetlands in Denmark are already located below the current mean sea level and lay behind dikes or pumping stations. This indicates an already high rate of coastal management/realignment which historically was set to increase land exploitation. Now the pattern is continuing, through the current climate adaptation plans, which seem widely to neglect the conservation of coastal habitats compared to urban development or infrastructure.

Even though the relative MSLR impacts on agricultural land are small (1.6%), we estimate that ~37,000 ha of current agricultural land will be below mean sea level by 2120, hence will need coastal protection via dikes or pumps if they are to remain profitable. Furthermore, a much larger agricultural area will be flooded due to storm surges. We project that under RPCP8.5 median MSLR 191,250 ha will be exposed to marine storm flooding by 2120 in Denmark. The increase in salinity in the soil might be a good starting point for gradually developing new coastal wetlands, if the agricultural activities are gradually to cease, perhaps as a mitigation measure for coastal wetlands conservation, within the national spatial plans. Given the nature of the Danish terrain, and the estimated mean MSLR, there is no doubt that marine flooding will be an issue of increasing concern. Failing to plan and implement measures that protect existing coastal habitats or allow their vertical and lateral expansion over time, within the national and local spatial plans, will be condemning them to extinction.

## Data Availability

Data is provided within the manuscript or supplementary information files. If further data is required, it can be obtain through communication with the corresponding author, Paula Canal-Vergés (canal@biology.sdu.dk)
